# A novel bioassay to detect Nociceptin/Orphanin FQ release from single human polymorphonuclear cells

**DOI:** 10.1371/journal.pone.0268868

**Published:** 2022-05-27

**Authors:** M. F. Bird, C. P. Hebbes, S. W. M. Scott, J. Willets, J. P. Thompson, D. G. Lambert

**Affiliations:** 1 Departments of Cardiovascular Sciences, University of Leicester, Anaesthesia, Critical Care and Pain Management, Leicester, United Kingdom; 2 Molecular and Cell Biology, University of Leicester, Anaesthesia, Critical Care and Pain Management, Leicester United Kingdom; Universite de Rouen, FRANCE

## Abstract

Nociceptin/Orphanin FQ (N/OFQ) is the endogenous opioid agonist for the N/OFQ receptor or NOP. This receptor system is involved in pain processing but also has a role in immune regulation. Indeed, polymorphonuclear cells (PMNs) express mRNA for N/OFQ precursor and are a potential source for circulating N/OFQ. Current measurements are based on ELISA and RIA techniques. In this study we have designed a bioassay to measure N/OFQ release from single PMNs. Chinese Hamster Ovary (CHO) cells transfected with the human (h) NOP receptor and Gα_iq5_ chimera force receptor coupling in biosensor cells to increase intracellular Ca^2+^; this can be measured with FLUO-4 dye. If isolated PMNs from healthy human volunteers are layered next to CHO_hNOPGαiq5_ biosensor cells then stimulated with the chemoattractant N-formyl-methionyl-leucyl-phenylalanine (fMLP) we hypothesise that released N/OFQ will activate the biosensor. PMNs also release ATP and CHO cells express purinergic receptors coupled to elevated Ca^2+^. In a system where these receptors (P2Y1, P2Y2 and P2X7) are blocked with high concentrations of PPADS and oATP, PMN stimulation with fMLP increases Ca^2+^ in PMNs then shortly afterwards the biosensor cells. Our data therfore reports detection of single cell N/OFQ release from immune cells. This was absent when cells were preincubated with the selective NOP antagonist; SB-612111. Collectively this is the first description of single cell N/OFQ release. We will deploy this assay with further purified individual cell types and use this to further study the role of the N/OFQ-NOP system in disease; in particular sepsis where there is strong evidence for increased levels of N/OFQ worsening outcome.

## Introduction

The Nociceptin opioid receptor (NOP) is the most recently discovered member of the opioid receptor family and is activated by an endogenous peptide agonist Nociceptin/Orphanin FQ (N/OFQ) [1]. Despite sharing a common transduction mechanism with classical opioid receptors, NOP has little or no affinity for ligands associated with classical naloxone-sensitive opioid receptors (mu:μ:MOP; delta:δ:DOP; kappa:κ:KOP). Importantly, actions at NOP are not sensitive to naloxone [1, 2]. Upon activation, NOP causes activation of the G_i/o_ G-proteins, leading to a decrease in cyclic AMP (cAMP) production, activation of inwardly rectifying potassium channels, closure of calcium channels and activation of the mitogen-activated protein kinase (MAPK) pathway. A significant body of research has focused on the role of NOP in analgesia and pain management. However, NOP is widely distributed in areas of the cardiovascular, immune, and central nervous systems, which suggest it is involved in a broad range of physiological processes [1–3].

NOP, N/OFQ and encoding mRNA have been detected in immune cells of both myeloid and lymphoid lineages [3, 4]. Evidence in patients with asthma, those undergoing surgery under cardiopulmonary bypass, and critically ill patients with sepsis suggest that N/OFQ-NOP may modulate acute inflammatory states, in which polymorphonuclear (PMN or granulocytes) cells play a significant role [3, 5–7]. This assertion is further supported by detection of transcripts encoding NOP and N/OFQ in polymorphonuclear leukocyte cells [6].

N/OFQ is increased in association with arthritis, asthma, sepsis and cardiopulmonary bypass in-vivo [6–8]. In an experimental caecal ligation/puncture model in rats, mortality was increased in animals also treated with exogenous N/OFQ, and mortality was decreased compared with controls in rats treated with the NOP antagonist UFP-101. These data suggest that N/OFQ increases in inflammatory states, and that it may have a modulatory effect on inflammatory processes [5].

However, the underlying source, and target for the observed increase in N/OFQ concentration is unclear. PMNs have a fundamental role in the response to bacterial infection, migrate to sites of inflammation when stimulated, contain NOFQ transcripts and secrete other substances [3]. PMNs are therefore a possible source.

**Fig 1:**
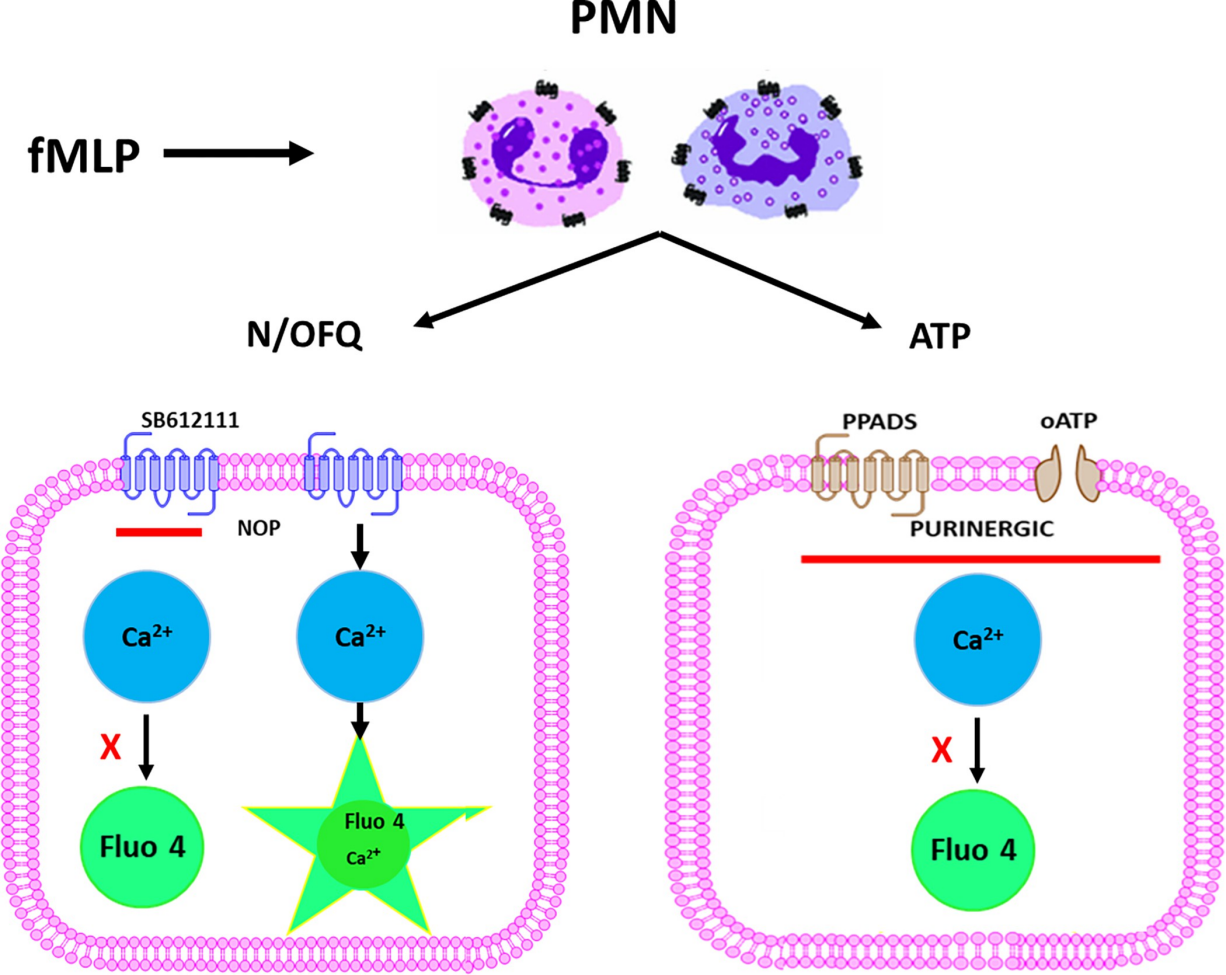
Schematic of representation of the immune cell bioassay. If immune cells produce N/OFQ peptide and release it in response to stimulation (with fMLP), released N/OFQ will bind to NOP receptors and chimeric G-protein on the surface of the CHO cell leading to an increase in calcium. This calcium increase can be measured using the fluorescent calcium indicator dye FLUO-4 and should be sensitive to NOP antagonists (SB-612111). CHO cells also express purinergic receptors that increase calcium in response to ATP also potentially released from PNMs; this can be blocked with purinergic antagonists (PPADS and oATP).

Capturing real-time release of N/OFQ in defined subpopulations of PMNs would improve understanding of its role in the immune response. We describe a method to demonstrate live-cell release of N/OFQ from cells, using mixed PMNs (that produce mRNA for N/OFQ precursor) as a model (**Fig 1**). This biosensor-based approach uses intracellular calcium concentration as a readout via an adapted human NOP receptor with a C-terminally modified Gα_qi5_ stably transfected into CHO cells (CHO_hNOPgαqi5_) [9]. If PMN degranulation releases N/OFQ from stores within defined PMN populations this will activate NOP in CHO_hNOPgαqi5_ to increase intracellular calcium concentration ([Ca^2+^]_i_). This can be observed in real time using the fluorescent calcium indicator FLUO-4. CHO cells are suited to this application because of the relative ease of transfection, and the existence of well characterised endogenous receptors. However, these cells are known to express purinoceptors [10, 11].

We hypothesised that a proportion of PNMs will release N/OFQ and this could be demonstrated using in CHO_hNOPgαqi5_ biosensor cells and an increase in [Ca^2+^]_i_. The aims of this work were to develop and validate this assay system.

## Materials and methods

### Extraction and isolation of PMN cells

Ethical approval was granted from the University of Leicester ethics committee (healthy volunteers). With written informed consent up to 30mls blood was collected from healthy volunteers into the S-Monovette collecting system (Sardstedt, Germany) containing K-EDTA (7.5ml blood per tube, final EDTA concentration 1.6 mg ml^-1^). Our donor pool comprised 16 participants, M:F = 9: 7 and age range 25–55. PMNs were extracted from blood within one hour of sampling by centrifugal separation over an equal volume of Polymorphprep (Axis-Shield, Dundee) as described previously [6]. The resulting PMN layer was removed, washed, cleared of erythrocytes by 1:1 dilution with BD PharmaLyse (Becton, Dickinson and Company, Oxford), washed and suspended in Krebs buffer (126 mM NaCl, 2.5 mM KCl, 25 mM NaHCO_3_, 1.2 mM NaH_2_PO_4_, 1.2 mM MgCl_2_, 2.5 mM CaCl_2_) for counting and imaging. Extractions were carried out at room temperature, and the resulting cell suspension maintained on ice until use (maximum 4 hours). Viability and yields were quantified by Trypan Blue exclusion and counting using a haemocytometer [12]. For determination of purity samples were further processed on a Becton-Dickinson FACS Aria II counter (BD Biosciences, San Jose, USA) with autoflurescence compensation. Final sample concentration was 1 x 10^6^ cells ml^-1^ prior to flow cytometry. PMN purity and identity data are presented in **S1 Fig in S1 File**.

### Cell culture

CHO cells transfected with the human NOP receptor and Gα_iq5_ chimera (CHO_hNOPGαiq5_) were a gift from Professor G. Calo (University of Ferrara, Italy; originally as a collaboration with Prof T. Costa, Instituto Superiore di Sanita, Rome, Italy). CHO_hNOPGαiq5_ were maintained in Hams 1:1 F12 Nutrient Mix and Dulbecos Minimal Essential Media (DMEM) (Invitrogen), supplemented with 10% Fetal Calf Serum, Penicillin (100 IU ml^-1^), Streptomycin (100 IU ml^-1^) and Amphotericin B (2.5g 10^−6^ ml^-1^). All cells were maintained in 5.0% CO_2_, humidified air, at 37°C, and routinely subcultured after 2–3 days when ≥ 90% confluence had been attained.

### Fluorimetric measurement of changes in CHO_hNOPGαiq5_ [Ca^2+^]_i_ in cell populations in response to N/OFQ

Fluorimetry was performed using a Perkin-Elmer LS50B fluorimeter (Beaconsfield, UK) using a standard Fura-2 based method as described [13]. Fluorescent emission was measured at 510 nm, with alternate excitation at 340 nm and 380 nm. Briefly, 2 mls of Fura-2 loaded cell suspension was introduced into a quartz cuvette and stirred using a magnetic stirrer, maintained at 37°C using a water jacket fed from a thermostatically-controlled water bath in the dark. Fluorescence was measured for 180 seconds prior to the introduction of 50 μl of test compound (40x in-assay concentration), and then until restabilisation of the signal. Following the final sample (per flask), the loading was calibrated to maximal and minimal fluorescence using 0.1% Triton X-100; 50 μl (Sigma Aldrich; Missouri, USA) followed by 4.5 x 10^−3^ M EGTA; 150 μl, pH>8 (Sigma Aldrich; Missouri, USA). Test compounds were maintained on ice, and cells were maintained at room temperature, protected from light throughout. Data were analysed using the fluorescence data manager (FLDM) software associated with the LS50B fluorimeter (Perkin-Elmer, Beaconsfield, UK). Raw fluorimetry data were processed using per-batch calibration, and values substituted into the Grynkiewicz equation [14] by FLDM software.

### Confocal measurement of changes in PMN and CHO_hNOPGαiq5_ fluorescence

28mm Number 1 coverslips (Thermo-scientific, UK) were sterilised in absolute ethanol, coated with CellTak™ tissue adhesive (Corning, New York USA) by adsorption, air dried, and stored at <5°C for a maximum of 7 days. Prepared coverslips were seeded with CHO_hNOPGαiq5_ (2.5–5 x 10^4^ cells per coverslip) and incubated as for cell culture for 18–24 hours before use. When ready for use, seeded coverslips were incubated with 6 x 10^−6^ M of the [Ca^2+^]_i_ dye FLUO-4 AM (Molecular Probes, Oregon USA), for 30–60 minutes at room temperature in the dark.

For microscopy, prepared coverslips were transferred to a PDMI-2 micro-incubator (Harvard Apparatus, Mass USA), and perfused at approx. 5 ml min^-1^ (4.81–4.96 ml min^-1^) with Krebs buffer at 36°C for 5-minute wash. In some experiments the layered coverslips were then incubated for 15 minutes in 5 x 10^−3^ M Pyridoxalphosphate-6-azophenyl-2’,4’-disulfonic acid (PPADS) and 8 x 10^−4^ M Adenosine 5′-triphosphate-2′,3′-dialdehyde (oATP) prior to imaging without further perfusion; this was to block endogenous purinoceptors. For NOP antagonist experiments, the NOP antagonist, 10^−6^ M SB-612111 was added at this point. During addition of PMNs or ligands, coverslips were imaged using a Nikon Eclipse T1Si inverted confocal laser scanning microscope, using Nikon EZC1 control software, excitation via a 488nm laser, and emission recorded at 513 – 556nm using a Nikon Apochromat-plan fluor oil immersion 60x objective. Time series were recorded at 2-second intervals. Gain and laser power were determined empirically.

Extracted PMNs were incubated with 6 x 10^−6^ M FLUO-4 AM at 36°C for 15 minutes, following which the cells were injected onto coverslips seeded with FLUO-4 AM loaded CHO_hNOPGαiq5_. Time sequences were recorded during application of the PMNs to the prepared coverslips (drop on method-injection of 100μl Krebs buffer containing requisite number of PMN directly onto coverslip), during fMLP (as a standard PMN secretagogue [15]) stimulation and until return to baseline. Baseline fluorescence and stimulated change in relative fluorescence of PMNs and CHO cells following fMLP treatment was assessed as described below.

Image analysis was undertaken in ImageJ, inbuilt Magic Montage tool and the BioFormats plugin. Regions of interest highlighting CHO_hNOPGαiq5_ cells and layered PMNs were determined manually. Changes in relative fluorescence were measured as described.

### Data analysis and statistics

#### Fluorimetric measurement of the change in [Ca^2+^]_i_ following ligand treatment in CHO cell populations

**Fig 2:**
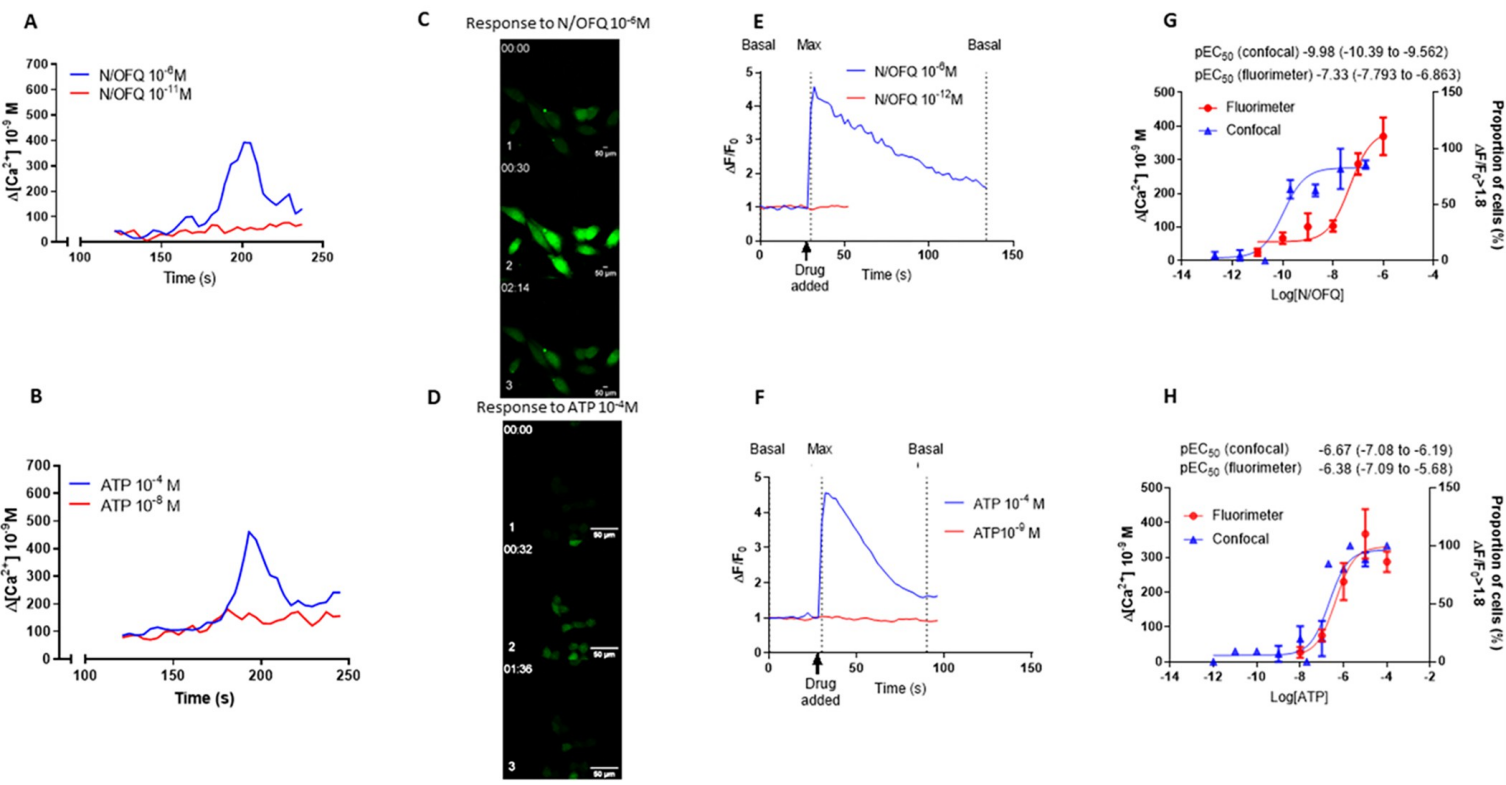
Fluoroscopy and microscopy response to agonists. A. Representative changes in intracellular [Ca^2+^] in response to exposure to N/OFQ as measured by cuvette based fluorimetry using FURA-2 as an indicator dye. B. Representative changes in intracellular [Ca^2+^] in response to exposure to ATP as measured by cuvette based fluorimetry using FURA-2 as an indicator dye. C. Representative change in ΔF/F_0_ in response to exposure to N/OFQ as measured by confocal microscopy using FLUO-4 as an indicator dye. (1. basal, 2. maximal, 3. decline). D. Representative change in ΔF/F_0_ in response to exposure to ATP as measured by confocal microscopy using FLUO-4 as an indicator dye (1. basal, 2. maximal, 3. decline). E. Representative change in ΔF/F_0_ in response to exposure to N/OFQ as measured by confocal microscopy using FLUO-4 as an indicator dye. F. Representative change in ΔF/F_0_ in response to exposure to ATP as measured by confocal microscopy using FLUO-4 as an indicator dye. G. Concentration-response curves showing maximal changes in intracellular [Ca^2+^] as measured by fluorimetry (red; n≥3), proportion of cells where maximal ΔF/F_0_≥1.8 (blue; total 1472 cells) as measured by confocal microscopy in response to stimulation by N/OFQ. H. Concentration-response curves showing maximal changes in intracellular [Ca^2+^] as measured by fluoroscopy (red; n≥3), proportion of cells where maximalΔF/F_0_≥1.8 (blue; total 51 cells) as measured by confocal microscopy. Error bars show SEM.

for each N/OFQ concentration, the Δ[Ca^2+^] was calculated by subtracting the mean of 3 baseline calcium concentrations from the maximal calcium concentration following the addition of the ligand. Log[Ligand]-[ΔCa^2+^]_i_ curve fitting was performed in GraphPad Prism V9 (22 October 2020). Experiments were performed in duplicate and repeated on ≥3 sets of cells from different passages. Normality was assessed using the D’Agostino–Pearson omnibus test. Representative curves are presented showing Δ[Ca^2+^]_i_ (e.g., **Fig 2A and 2B**). Concentration-response curves are presented as mean ± SEM (e.g., **Fig 2G and 2H**). Summary LogEC_50_ data are presented as mean (CI95).

#### Single cell measurement of change in [Ca^2+^]_i_

thresholding was used to highlight areas of increased intensity, segmented into regions of interest to represent cells. The coordinates of these defined regions of interest (ROI) are superimposed to the original image, optimised by hand, and used to measure mean fluorescent intensity within each cell. For each cell of a given cell type (for example, PMNs or CHO cells), mean fluorescence (F) was measured at 2 second intervals. ΔF/F_0_ was determined at each timepoint (where ΔF is F-F_0_, and F_0_ is the basal fluorescence, the mean fluorescence for the first 5 images for each region of interest). Cells with ΔF/F_0_>1.8 were classified as responsive based on a high sensitivity and low false positive rate (**see S2-S5 Figs and S1-S3 Tables in S1 File for detailed analysis methodology**). When analysing concentration-responses by confocal microscopy the ΔF/F_0_ > 1.8 cut-off and GraphPad Prizm were used. Statistical tests are noted in the figure legends with significance determined as p<0.05.

## Results

First we needed to examine responsiveness of our biosensor CHO_hNOPGαqi5_ cells to (i) N/OFQ as our primary end point and the peptide we intended to assay and (ii) ATP as an identified endogenous transmitter for which CHO cells have target receptors linked to increases in [Ca^2+^]_i_.

Cuvette based fluorimetry demonstrates a monophasic increase in response to N/OFQ (**Fig 2A**) and ATP (**Fig 2B**). The response to N/OFQ was concentration dependent with a pEC_50_ of 7.33 (95%CI 7.79–6.86) and maximal response at 10^-6^M (**Fig 2G**).The increase in response to ATP was also concentration dependent with a pEC_50_ of 6.38 (5.68–7.09) and maximal response at 10^−6^ M (**Fig 2H**).

There was an increase in mean fluorescence of CHO_hNOPGαiq5_ cells detected by confocal fluorescence microscopy following stimulation by N/OFQ (**Fig 2C and 2E**) and this was concentration dependent, with a pEC_50_ of 9.98 (9.56–10.39) and maximum at ~10^−8^ M N/OFQ (**Fig 2G**). There was a similar increase with ATP (**Fig 2D and 2F**) yielding a pEC_50_ of 6.67 (6.19–7.08) and maximum at ~3 x 10^−6^ M ATP (**Fig 2H**). There was a significant difference between pEC_50_ for N/OFQ in cuvette and confocal systems with adherent cells in the confocal displaying a more potent response. As controls CHO_wt_ cells did not respond to N/OFQ and CHO_hNOPGαqi5_ biosensor cells did not respond to fMLP (**S6 Fig in S1 File**).

**Fig 3:**
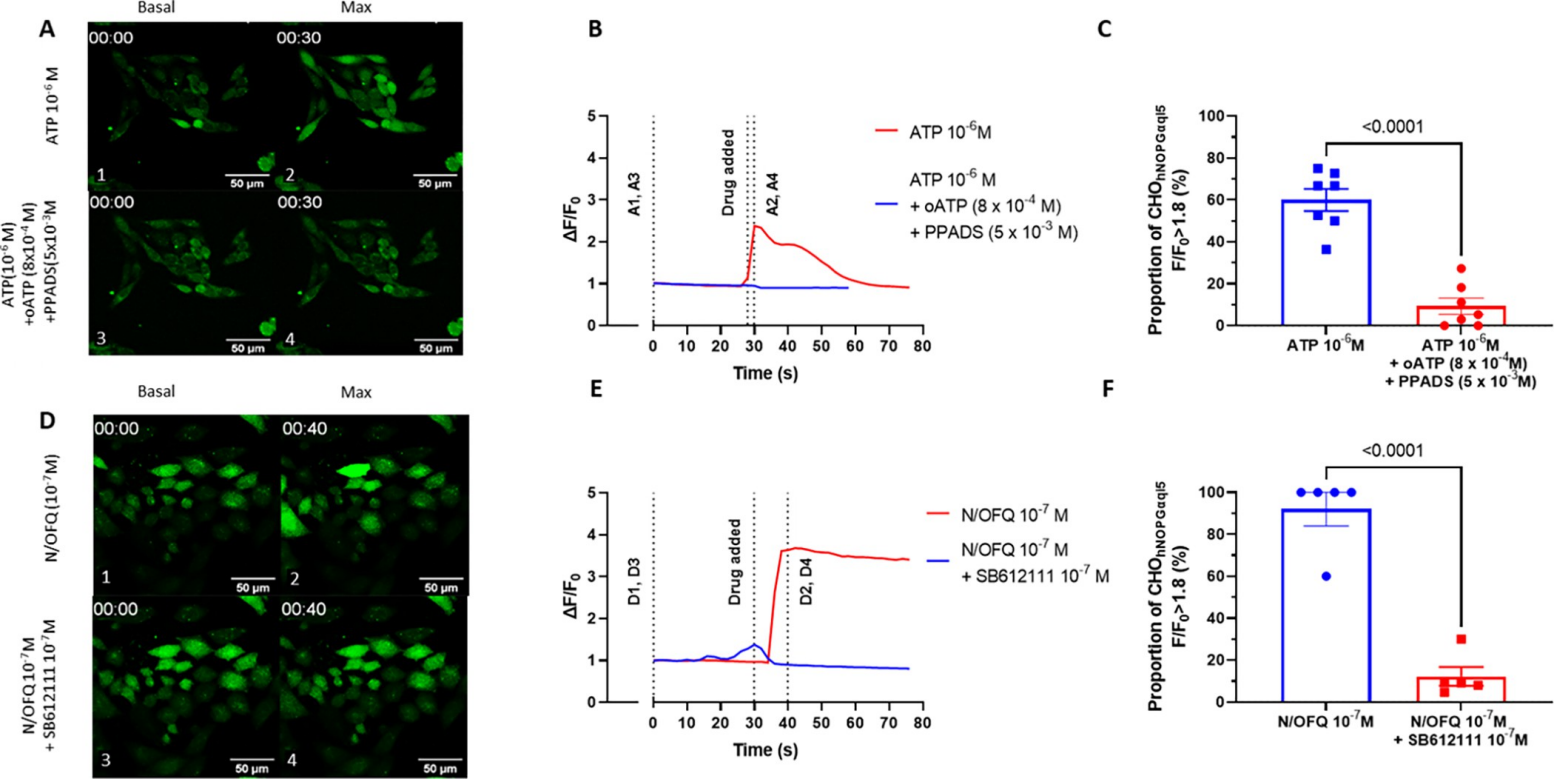
Response of CHO_hNOPGαqi5_ to ATP and N/OFQ in the presence and absence of antagonists. *A*. Representative change in ΔF/F_0_ in response to exposure to 1x10^-6^ M ATP in the absence (1, 2), and presence (3, 4) of the purinergic antagonists PPADS 5x10^-3^ M and oATP 8 x10^-4^ M as measured by confocal microscopy using FLUO-4 as an indicator dye. (basal 1, 3; maximal, 2, 4). *B*. Representative change in ΔF/F_0_ in response to exposure to 1 x10^-6^ M ATP as measured by confocal microscopy using FLUO-4 as an indicator dye in the presence and absence of PPADS and oATP in the concentrations stated. *C*. Combined cellular responses following exposure to 1 x10^6^ M ATP in the presence and absence of PPADS and oATP in the concentrations stated. *D*. Representative change in ΔF/F_0_ in response to exposure to 1 x10^-7^ M N/OFQ in the absence (1, 2), and presence (3, 4) of the antagonist SB-612111 1 x10^-7^ M as measured by confocal microscopy using FLUO-4 as an indicator dye. (basal 1, 3; maximal, 2, 4). *E*. Representative change in ΔF/F_0_ in response to exposure to 1 x10^-7^ M N/OFQ as measured by confocal microscopy using FLUO-4 as an indicator dye in the presence and absence of 1x10^-7^M SB-612,111. *F*. Combined cellular responses following exposure to 1 x10^-7^ M N/OFQ in the presence and absence of 1x10^-7^M SB-612,111. Error bars where shown are SEM (n = 7), p values from unpaired t-test.

As noted, PMNs release ATP so we next needed to confirm that we could block the effects of this transmitter in the confocal system. The response to ATP can be antagonised by the purinergic antagonists PPADS and oATP, targeted at the P2Y1, P2Y2 and P2X7 receptors. The optimal concentrations of PPADS and oATP were determined empirically (**S7 Fig in S1 File**); we elected to use 5 x 10^−3^ M PPADS and 8 x 10^−4^ M oATP. This combination of antagonists blocked the response of CHO_hNOPGαqi5_ cells to 10^−6^ M exogenous ATP (**Fig 3A–3C**). The proportion of responding cells (**Fig 3C**) was significantly reduced by the combined antagonists. Next we needed to know if the response to exogenously added N/OFQ could be blocked by the NOP antagonist SB-612111. In **Fig 3D–3F** the response to N/OFQ was blocked by coincubation with 10^-7^M SB-612111, demonstrating the antagonist ability of this ligand.

These data confirm that CHO_hNOPGαqi5_ is a SB-612111 sensitive detector for N/OFQ; there is also a significant response to exogenous ATP which can be antagonised by coincubation with the purinergic antagonists PPADS and oATP under the stated conditions.

**Fig 4:**
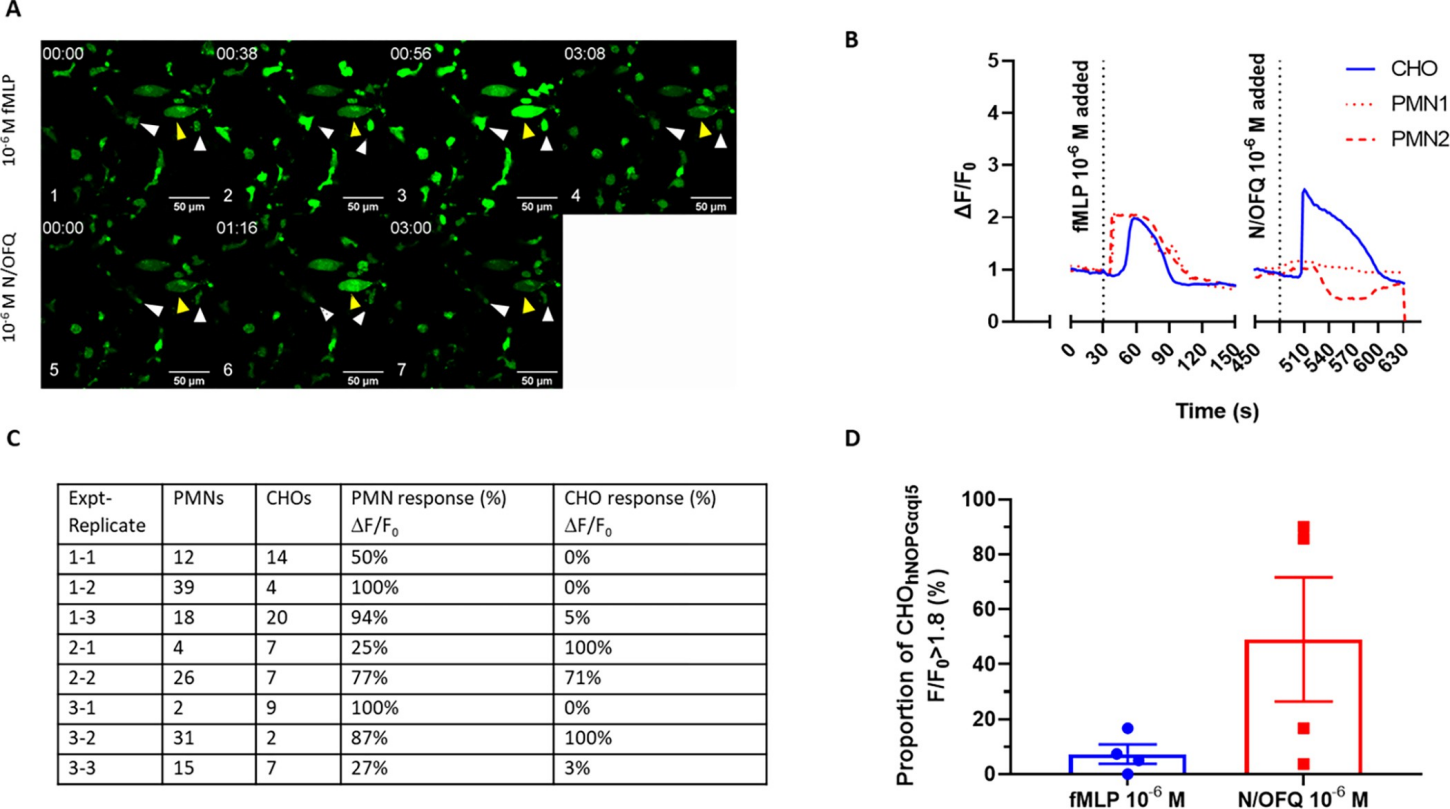
Responses of CHO_hNOPGαqi5_ to 10^-5^M fMLP and 10^-6^M N/OFQ. *A*. Representative change in ΔF/F_0_ of CHO with colayered PMNs in response to exposure to 1 x10^-6^ M fMLP (1–4) and 1 x10^-6^ M N/OFQ (5–8) as measured by confocal microscopy using FLUO-4 as an indicator dye. Basal 1, 5; maximal PMN, 2; maximal CHO 3, 6; return to basal, 4, 7). PMNs are identified by white arrows, while CHOhNOP_Gαqi5_ are identified by yellow arrows. *B*. Representative single experiment showing change in ΔF/F_0_ of CHO cells with colayered PMNs in response to exposure to 1 x10^-6^ M fMLP, washing and 1 x10^-6^ M N/OFQ as measured by confocal microscopy using FLUO-4 as an indicator dye. *C*. Proportion of colayered PMN and CHO cells responsive following exposure to 1 x10^-6^ M fMLP. *D*. Combined CHO cellular responses following exposure of colayered PMNs to 1 x10^-6^ M fMLP, washing and then 1 x10^-6^ M N/OFQ. Error bars show SEM (n = 4).

Next we colayered PMNs and CHO cells. As shown in **Fig 4A and 4B**, following exposure to 10^−6^ M fMLP, first PMNs (identified by white arrows) and then adjacent CHO cells (identified by a yellow arrow) showed an increase in relative fluorescence, comparable to individual responses from exposure to 10^−6^ M N/OFQ, suggesting an increase in intracellular calcium from NOP receptor activation. The CHO response following fMLP stimulation of colayered PMNs (**Fig 4C**) was lower than the CHO response after exposure to 10^−6^ M exogenous N/OFQ (**Fig 4D**). Collectively these data indicate that some of the PMNs were releasing something that is stimulating the biosensor CHO cells. Based on the above experiments with ATP we then investigaed whether there was a purinergic signal.

**Fig 5:**
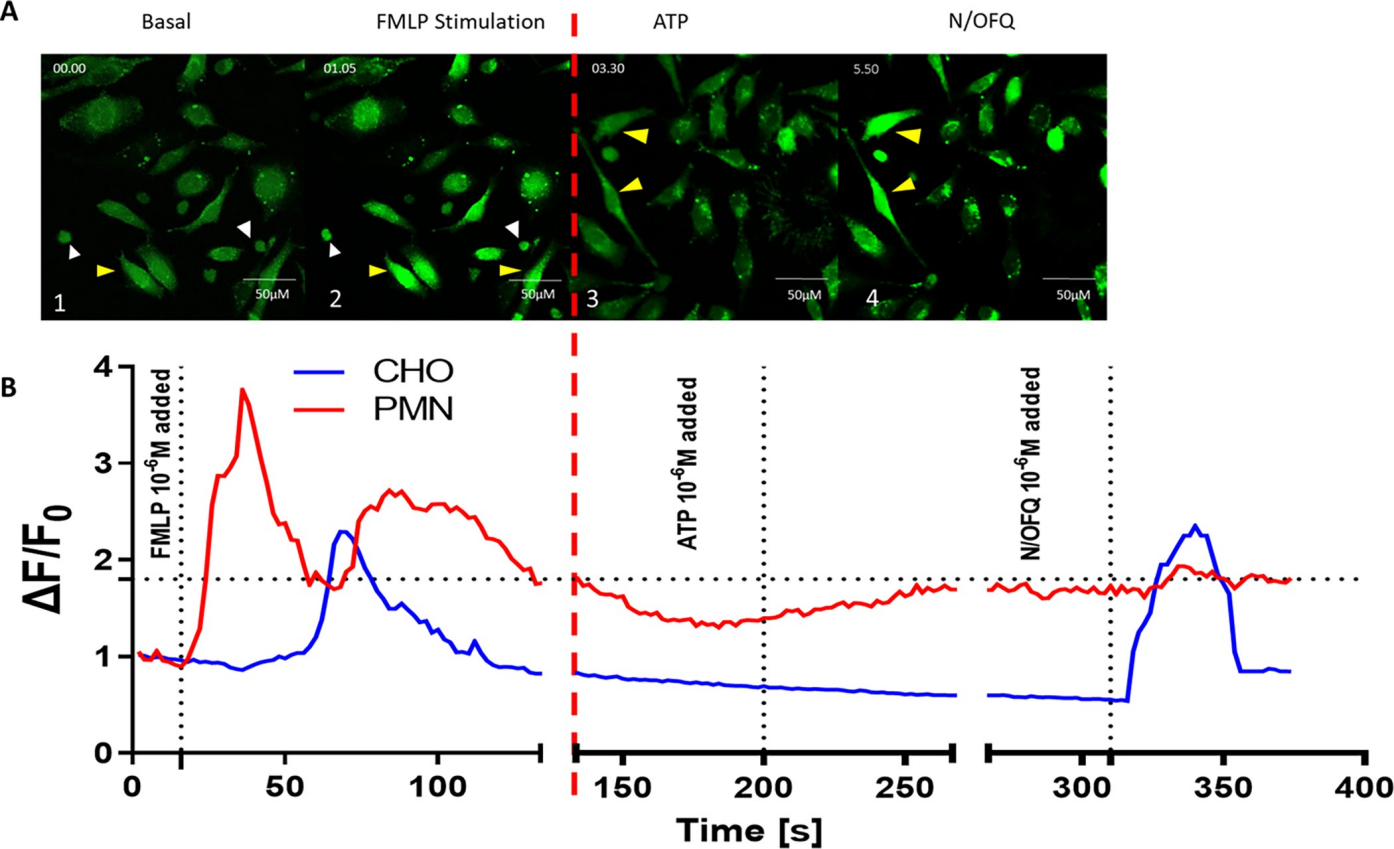
CHO_hNOPGαqi5_ response to PMN degranulation stimulated by fMLP 1 x10^-6^ M in the presence of PPADS (8 x 10^−4^ M) and oATP (5 x 10^-3^M). A. Representative micrograph (from n = 6) depicting 1) basal activity, 2) fMLP stimulation of PMN and subsequent activation of CHO_hNOPgq/i5_ (with representative samples of labelled CHO (yellow) and PMN (white)), 3) addition of 1x10^-6^ ATP as a purinergic challenge and, finally, 4) N/OFQ to demonstrate functional viability of the CHO cells. B. Associated graph demonstrating change in ΔF/F_0_ following treatment with fMLP, ATP and N/OFQ. Dotted red line indicates a change of field on the same coverslip to avoid photobleaching due to the length of the experiments.

When coincubated PMNs (identified by white arrows) and CHO cells (identified by yellow arrows) were exposed to 10^−6^ M fMLP in the presence of 8 x 10^−4^ M oATP and 5 x 10^−3^ M PPADS, there was initiallly stimulation of PMNs followed by a delayed activation of CHO cells (**Fig 5A and 5B**). These data indicate that the response in the biosensor cells was not caused by ATP release.

**Fig 6:**
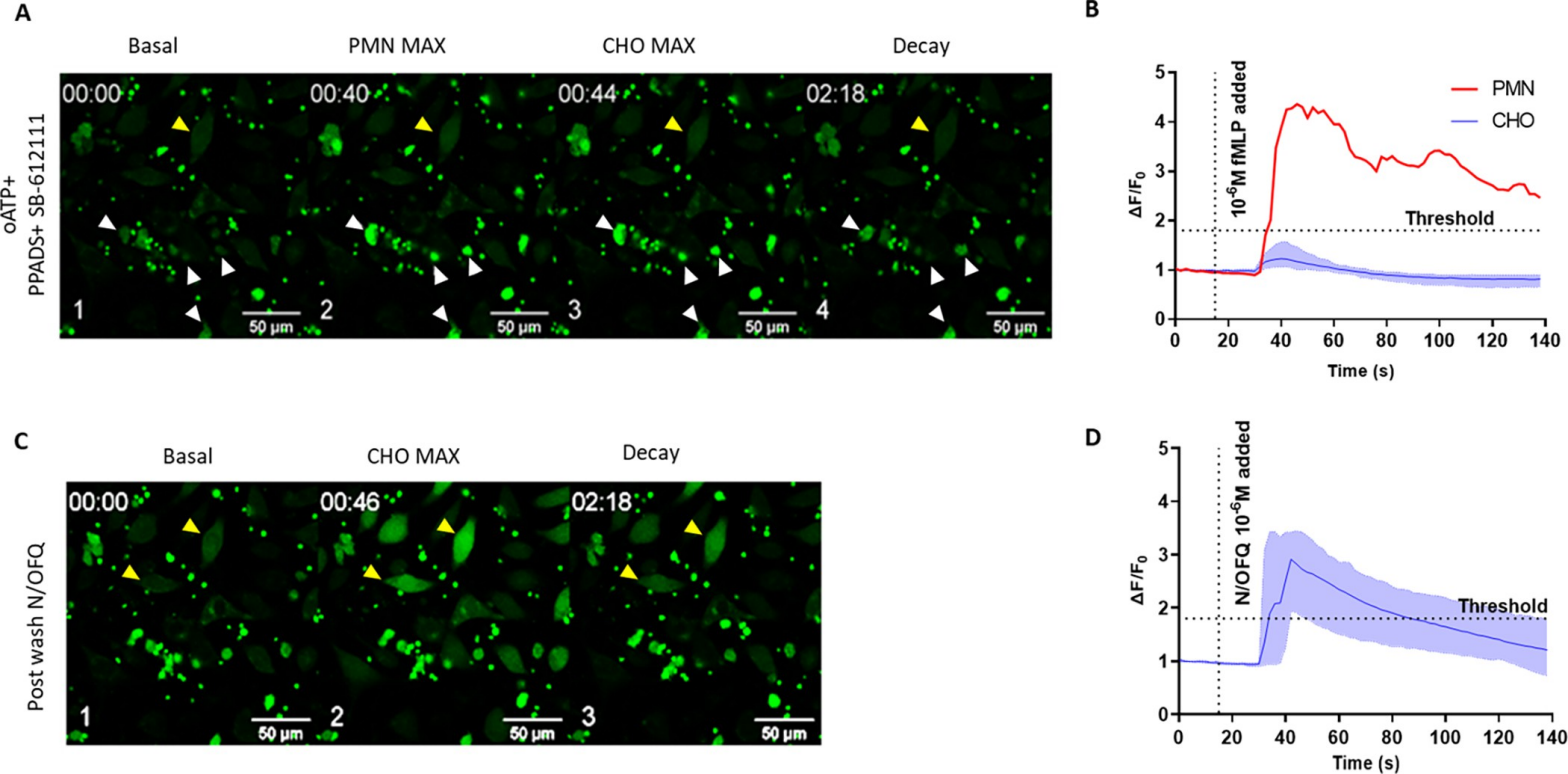
CHO_hNOPGαqi5_ colayered with PMNs stimulated with fMLP 10^-6^M in the presence of PPADS, oATP and SB-612111 do not increase calcium. A. Representative photomicrograph (from n = 6) showing 1. Basal, 2. Maximal PMN, 3. Maximal CHO stimulation in response to 1x10^-6^M fMLP in the presence of PPADS, oATP and SB-612111 measured by confocal microscopy using FLUO-4 as an indicator dye. PMNs are identified by white arrows, while CHO_hNOPGαqi5_ are identified by yellow arrows. B shows corresponding ΔF/F_0_ over time; shaded areas denote minimal and maximal response for the cell population. C. Representative photomicrograph (from n = 6) showing 1. Basal, 2. Maximal CHO stimulation in response to N/OFQ 1 x10^-6^ M in the absence of antagonists. D shows corresponding ΔF/F_0_ over time and return of N/OFQ response; shaded areas denote minimal and maximal response for the cell population.

Using a final protocol (with PPADS and oATP and SB-612111) of layering PMNs (identified by white arrows) onto CHO biosensor cells (identified by yellow arrows) followed by 15-minute incubation and a 10-minute wash, treatment with fMLP led to an increase in PMN but not CHO fluorescence (**Fig 6A and 6B**). Cells remained viable and responded to N/OFQ following washing of the antagonists (**Fig 6C and 6D**).

In summary, these data demonstrate a specific, repeatable increase in relative fluorescence in response to N/OFQ released from the degranulation of a proportion of the mixed PMN population. This was single cell N/OFQ release.

## Discussion

The basis of this work was to identify a suitable system to detect release of N/OFQ from isolated single PMNs. Forced N/OFQ-dependent increases in [Ca^2+^]_i_ have been well documented in CHO_hNOPgαi/q_ cells using traditional fluorescence-based calcium mobilisation cuvette or microplate assays [9] and, therefore, represents the best translatable model for confocal based measurement of potential N/OFQ release.

Our initial experiments involved comparing concentration-responses for calcium increase and proportion of responsive (maximal F/F_0_ > 1.8) cells after N/OFQ exposure in cuvette and confocal assays respectively. The estimated pEC_50_ values were 7.3 and 10.0 for the cuvette and confocal assays respectively. Previous published pEC_50_ values for N/OFQ in this cell line, in a microplate FLUO-4 based assay, was 9.09 [9]. There is a 500 fold difference in potency between the cuvette and confocal assay in our hands. There was also a difference between confocal and microplate potency values of around 10 fold. The lowest concentration of N/OFQ at which we were able to detect a confocal response was 100pM. It should be noted that cuvette based assays involved cells in suspension using Fura-2 while confocal assays involved measurement of adherent cells using FLUO-4. The use of different dyes is unlikely to explain the difference and we feel this is due to use of adherent cells over suspensions. In suspensions of cells using a stirred system, peptidase enzymes are more likely to be released or present in cell debris effectively metabolising and reducing N/OFQ concentrations; experiments with peptidase inhibitors would confirm this supposition. Comparing microplate and confocal data the latter uses a perfusion protocol which will most likely clear the bath of enzyme activity. The lack of difference with the non-peptide ATP (below) in the two systems adds weight to our explanation. Importantly, our data indicate we can detect exogenously added N/OFQ to sub 100pM concentrations. This equates to ~181pg ml^-1^. In a previous publication we summarised plasma N/OFQ concentrations in a range of assays over a range of diseases at 2.3–172 pg ml^-1^. Clearly these are in total circulating volume but guide effect site ranges [16].

As noted earlier, ATP is present in PMN cells [17–19]. ATP is a damage-associated molecular pattern (DAMP) molecule, and is associated with PMN chemoattraction, phagocytosis and apoptosis. This activity in PMNs is believed to occur through ATP-sensitive, ligand-gated ion channel P2X7 receptors, with the receptor being present on lymphocytes, monocytes and macrophages [20–24]. ATP release is believed to occur when immune cells are activated [22], and as the process of cell isolation is likely to provide significant activation, ATP release is expected in these experiments. CHO cells do not express high numbers of native receptor types, making them an ideal cell line for experimentation. However, they do express two types of purinergic receptors, the P2X7 [11] and P2Y2 [10]. P2Y2 receptors are G_q_-coupled GPCRs, whose activation also leads to increases in calcium levels. Most importantly, with regards to the development of the bioassay, activation of these purinergic receptors potentially masks any N/OFQ activity in the chimeric CHO model readout.

CHO_hNOPGαqi5_ cells consequently have the machinery to respond to both N/OFQ and ATP; the former due to active transfection and the latter due to the expression of the noted endogenous purinoceptors. Effective antagonism of the purinergic signal is critical to interpretation of our data and ascription of a rise in biosensor Ca^2+^ to N/OFQ and not ATP. Antgonist concentrations were chosen after a series of challenge experiments using 1x10^-6^M ATP. These experiments indicated that a combination of 5x10^-3^M PPADS and 8x10^-4^M oATP blocked ATP-induced calcium increase (**S7 Fig in S1 File**), indicating that any remaining calcium response in the presence of these antagonists is not via purinergic signalling.

In both confocal and fluorometric experiments, ATP produced similar pEC_50_ of 6.67 and 6.37 respectively. The non-peptide nature of this mediator supports our explanation above of the wide variation in N/OFQ potency in the two assay formats. Using a luciferase based assay we determined PMN ATP contents in the μM range; released contents would be lower but are likely comparable to our EC_50_ values of ~0.2–0.4μM (**S8 Fig in S1 File**). Reported plasma ATP concentrations vary widely with sample preparation being an important consideration. A value of 1μM; higher around the releasing immune cells (the putative effect site) is not unreasonable [23].

In order to confirm NOP activation we have used the NOP antagonist SB-612111 [25, 26]. In our hands this antagonist has high affinity and high selectivity for NOP. In CHO cells expressing recombinant human NOP, MOP, DOP and KOP receptors SB-612111 dsiplayed K_i_ (binding affinity) values of 0.7, >1000, >5000 and >1000 respectively. Moreover, GTPγ[^35^S] binding (membranes) and cAMP (whole cells) assays at human NOP indicate antagonist K_B_ values of 0.2 and 2.3nM respectively [25]. In the present study we used SB-612111 at the fixed concentration of 1x10^-7^ to ensure selectivity of action.

We selected fMLP as our PMN ‘secretagogue’. This is a simple three amino acid (n-formyl-methionyl-leucyl-phenalanine) peptide [15]. There are three formylpeptide receptors [27] and fMLP interacts with FPR1 coupled to G_i/o_ and G_z_ [28]. G_iβ/γ_ stimulates phospholipase-C and phosphoinositide 3-kinase to both increase [Ca^2+^]_i_ and activate MAPK [29, 30]. In immune cells such as PMNs this results in a wide range of actions including degranulation and chemotaxis. Indeed, degranulation is the main reason for use in this work; as an N/OFQ ‘secretagogue’. In our confocal experiments we demonstrated a robust increase in Ca^2+^ and this precedes activation of CHO biosensor cells.

In our initial experiments, we measured PMN stimulation with fMLP to determine whether CHO_hNOPGαqi5_ cells survived in the presence of these freshly isolated immune cells and whether a response to immune cell stimulation could be detected on the confocal microscope. Addition of fMLP led to an initial rise in calcium in immune cells, associated with peptide release, and following a delay, an increase in fluorescence in the CHO cells. This delayed response would indicate that substances released from immune cells has triggered a calcium response in CHO cells. In order to mitigate any potential responses produced by ATP released from immune cells the pre characterised purinergic receptor antagonist combination was added. Following fMLP stimulation, a delayed activation of CHO_hNOPGαqi5_ was still present, indicating the involvement of immune cell released N/OFQ. Interestingly, only some CHO_hNOPGαqi5_ responded following fMLP stimulation. When assessing single cell CHO_hNOPGαqi5_ responses when coincubated with PMNs and stimulated with fMLP, there is a clear temporal relationship between local PMN and CHO response. As this is a mixed population of PMN cells, it is possible that some but not all cells release N/OFQ following stimulation.

In a previous series of experiments we demonstrated that NOP mRNA was expressed in all immune cell populations. The presence of NOP suggests a target for feedback loops and the differential expression of N/OFQ precursor (ppN/OFQ) provides a mechanism for cell-cell interaction. With respect to the current experiments differenatial expression of ppN/OFQ and the presumed potential to release N/OFQ could explain the variability in biosensor response. Simple proximity (effect site dimensions) could also be in play.

In order to confirm whether NOP receptor activation occurs on CHO_hNOPGαqi5_ during this process, the NOP antagonist SB-612111 was preincubated on CHO cells with oATP and PPADS. Following addition of PMN cells and stimulation by fMLP, no activation above threshold of the CHO_hNOPGαqi5_ was seen, indicating that blockade of the NOP receptor has inhibited the calcium-induced increase in fluorescence. **This indicates that N/OFQ is being released from live PMN cells following stimulation**.

While it is widely accepted that N/OFQ is present within immune cells, understanding of its release is limited. In their important work studying the role of leukocytes in pain, Rittner and colleagues used flow cytometry to measure overall changes in opioid peptide expression in immune cells [31]. Following administration over several timepoints with Freuds adjuvant, a pan-opioid peptide antibody, called 3E7, detected opioid peptides amongst several immune cell subtypes. Furthermore, beta-endorphin containing cells were collected via magnetic sorting and immunohistochemistry, with the levels of beta-endorphin measured by radioimmunoassay. From these results, it was determined that in the early stages PMNs were the primary opioid-producing cells, while at later timepoints monoctyes and macrophages became the predominant opioid-containing cells. While these experiments do demonstrate that immune cells contain opioid peptides, they do not demonstrate peptide release, or what potential condition cells are in prior to opioid release. Moreover, this study focussed on classical opioids and not the N/OFQ system.

For the involvement of N/OFQ in the immune system in particular, work has either focussed on circulating N/OFQ or using non-human tissue. Previous work from our laboratory demonstrated a significant rise in circulating N/OFQ in patients with sepsis, however the source(s) were not determined [6]. N/OFQ has been linked with arthtiritis, potentially with secretion from PMN’s in the synovial fluid [8]. In this study, several experiments where undertaken to detect N/OFQ ranging from ELISA to mass spectrometry. From these results it was determined that PMN’s contain N/OFQ [8]. These results compliment our findings. Our suggestion that in healthy volunteers not all PMNs secrete N/OFQ is a further refinement.

Whilst this work does demonstrate a release of N/OFQ from PMN cells our process is not without drawbacks. The most important being the process of immune cell extraction itself. This is likely to represent a major stimulus and may induce activation and degraulation thereby reducing the pool of cells capable of release. That said extracted immune cells displayed high degrees of viability and fMLP experiments showed that the vast majority of immune cells were capable of responding (as a rise in Ca^2+^) to an attractant stimulus. We have been as gentle as possible in our preparatory phase and also a rapid as possible in the isolation phase. The immune cells we are using, ex vivo, from volunteers are assumed to represent the situation of PMNs in the general circulation and at rest; this may not be the case. In addition, as all immune cell types express NOP mRNA then interaction between cell types is something that could occur in vivo that we have not recreated in this bioassay.

In summary. we have designed, developed and validated a new confocal microscopy-based bioassay to detect N/OFQ release from live single immune cells. Using this assay, we have demonstrated the first evidence of live release of N/OFQ from PMNs. We plan to use this assay to explore release from individually identified immune cells (using standard immune markers) and to deploy this assay in samples from critically ill patients with sepsis.

## Supporting information

S1 File (DOCX)
