# Supplementary material for: A novel bioassay to detect Nociceptin/Orphanin FQ release from single human polymorphonuclear cells
Source: PLoS One. 2022 May 27;17(5):e0268868. doi: 10.1371/journal.pone.0268868 (PMC9140256; doi:10.1371/journal.pone.0268868)
Supplement: S1 File — (DOCX) [file pone.0268868.s001.docx]

**A Novel Bioassay to Detect Nociceptin/Orphanin FQ Release from Single Human Poylmorphonuclear Cells**

^1^Bird MF, ^1^Hebbes CP, ^1^Scott SWM, ^2^Willetts J, ^1^Thompson JP and ^1*^Lambert DG^1*^.

***MFB and CPH are joint first authors.***

^1^Departments of Cardiovascular Sciences and ^2^ Molecular and Cell Biology, University of Leicester, Anaesthesia, Critical Care and Pain Management, Hodgkin Building, Leicester, LE1 9HN. UK.

**SUPPORTING INFORMATION; SUPPLEMENT.**

**1. Assessment of PMN purity and identity**

Ethical approval for this work was granted by the University of Leicester ethics committee (UOL0554). Written consent was obtained from all volunteers.

Immunocytes were extracted from whole blood, and cleared of erythrocytes. Blood was sampled from healthy volunteers (up to 30ml volumes from a pool of 16 participants, M:F = 9: 7 and age range 25-55) into Monovette 7.5ml collection tubes containing K3-EDTA (Sarstadt Monovette K3E 01.1605.004) and mixed well before extraction of mixed PMNs.

Mixed PMNs were obtained as detailed in main manuscript by centrifugal separation over an equal volume of polymorphprep (Axis-Shield, Dundee).

Resultant preparation was diluted in sterile, filtered phosphate buffered saline, counted by haemocytometry and stained.

Antibodies directed against CD66, Siglec-8 and CD45 and conjugated to the fluorophores Fluorescein isothiocyanate (FITC), Allophycocyanin (APC), and Phycoerythrin (PE) were used to verify gating and quantify numbers of granulocytes, eosinophils and leucocytes respectively. Cytometry data were exported and analysed using FCSAlyzer 0.9.15 software. Aggregate data were analysed using GraphPad Prism. Viability and purity are presented as mean % ± standard deviation. Yield is presented as total cells ml^-1^ whole blood.

**2. Verification and validation of PMN s**

Gates were set for cell populations based on both physical characteristics (FSC/SSC), and the expression of cell surface markers **(Supplement Table 1)**. Granulocytes were detected based on the exclusion of debris by FSC/SSC and detection of CD66.

| **Cell type** | **Strategy** | **Positive control** | **Negative control** |
| --- | --- | --- | --- |
| Cells vs Debris | FSC/SSC | Whole blood |  |
| Granulocytes | FSC/SSC, CD66abce | Mixed PMN | Unstained Monocytes |

**Supplement Table 1:** Determination of PMN subtypes and the methods used to validate findings. Flow cytometry gating strategy.

PMN purity data are presented in **Supplement Figure-1**. From these experiments we determined that 81.76% of the detected material following polymorphprep separation represented CD66+ granulocytes.


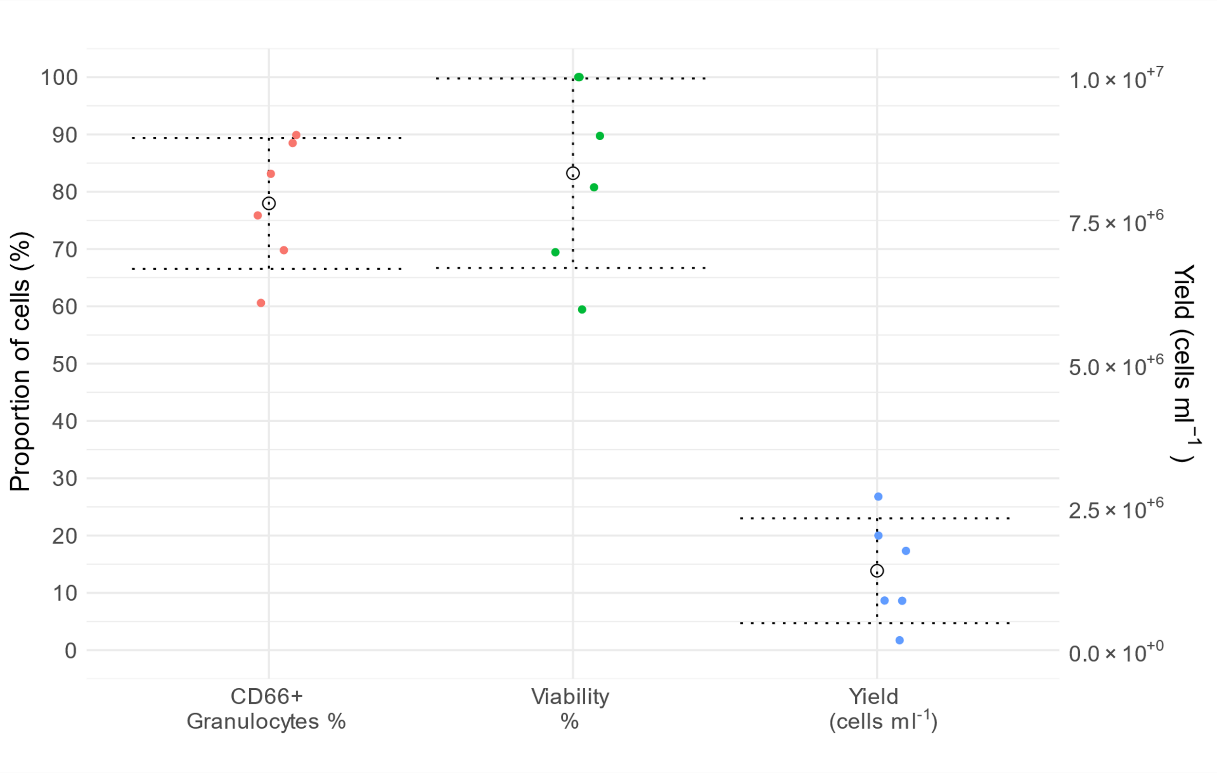


A – Purity, viability and yield of granulocyte separation by Polymorphprep™ - Error bars represent mean ± SD
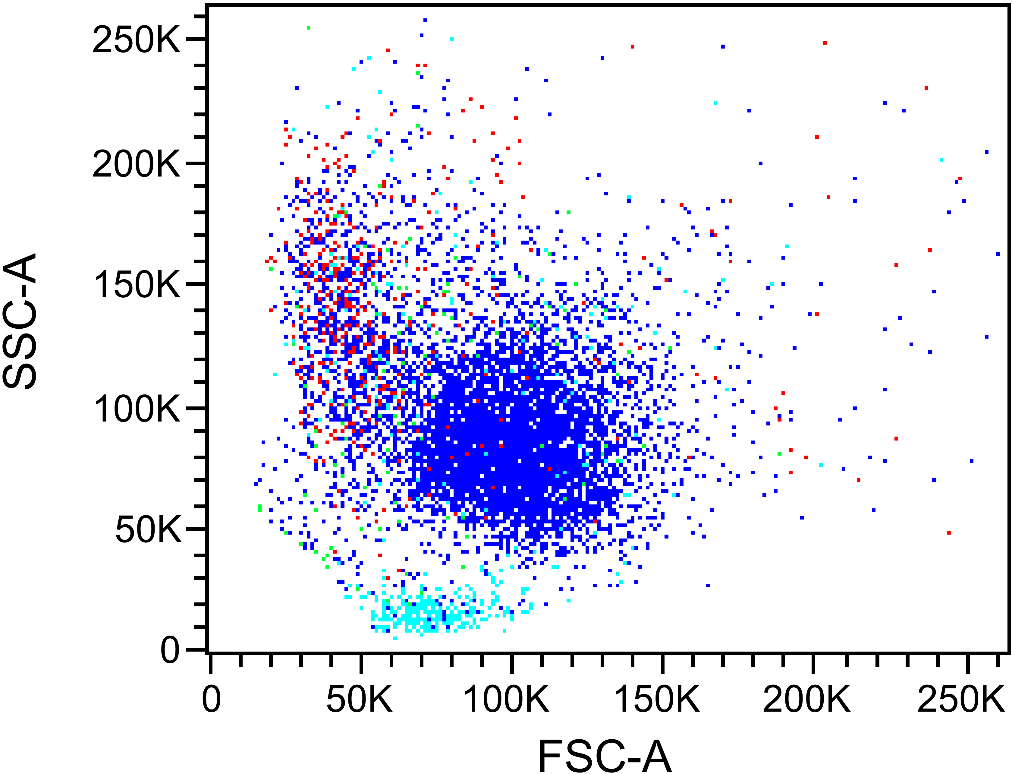


B – Representative triple stained dot plot gated (FSC/SSC) for nucleated cells showing leucocytes (pale blue), eosinophils(red) and granulocytes (dark blue). Doublets/Triplets not discriminated

**Supplement Figure 1**: Identity and purity of separated PMNs

**3. Strategy for setting response threshold for single cells**

Across 5 experiments, the overall variation in maximal F/F_0_ for all cells is shown when treated with 10^-6^ M N/OFQ and buffer **(Supplement Figure-2 and 3)**.


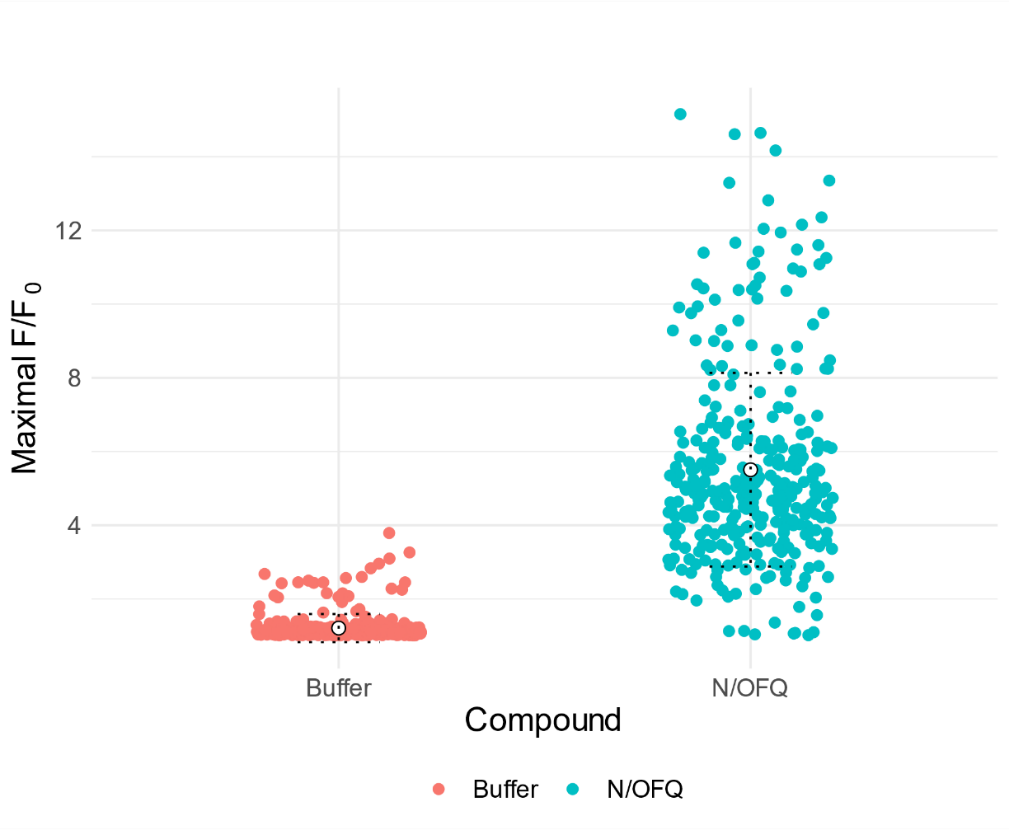


**Supplement Figure 2:** Maximal F/F_0_ for every cell after treatment with 10^-6^M N/OFQ and buffer

- n=5 experiments, total 343 cells, p<0.05 (independent samples t-test). Error bars denote mean ± SD

Using buffer as a negative control, and N/OFQ as a positive control, the separation of maximal F/F_0_ values are shown with the percentiles **(Supplement Table-2)**.

|  | **% cells below threshold F/F_0_** | | | |
| --- | --- | --- | --- | --- |
|  | **25%** | **50%** | **75%** | **95%** |
| **10^-6^M N/OFQ** | 3.88 | 4.93 | 6.26 | 11.11 |
| **Buffer** | 1.05 | 1.09 | 1.17 | 2.15 |

**Supplement Table 2:** Percentiles for maximal F/F_0_

for a prototypical positive control, 100 μL N/OFQ (final concentration 10^-6^M), and a negative control, 100 μL buffer.


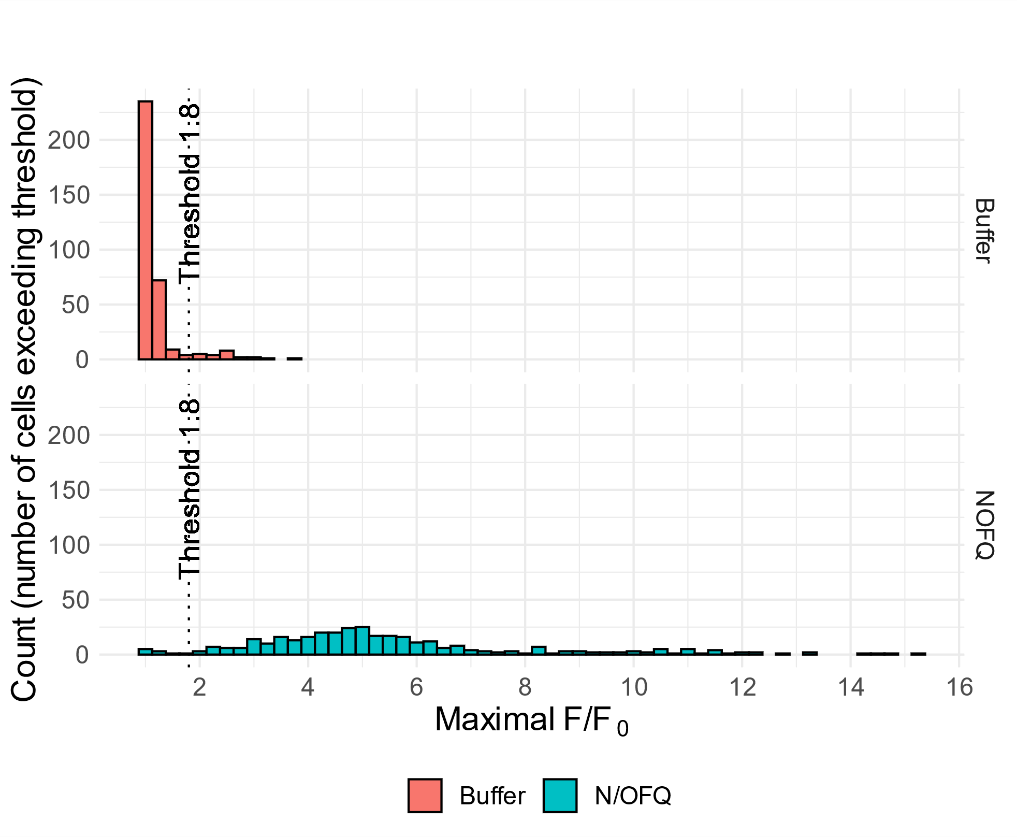


**Supplement Figure 3:** Frequency distribution of maximal relative fluorescence following treatment of CHO_hNOPGαqi5_ with 10^-6^M N/OFQ and buffer

- The threshold point of F/F_0_ ≥ 1.8 is shown.

Receiver-Operating Characteristic (ROC) analysis of these data indicate the optimal threshold for discrimination. **(Supplement Figure 4)**.


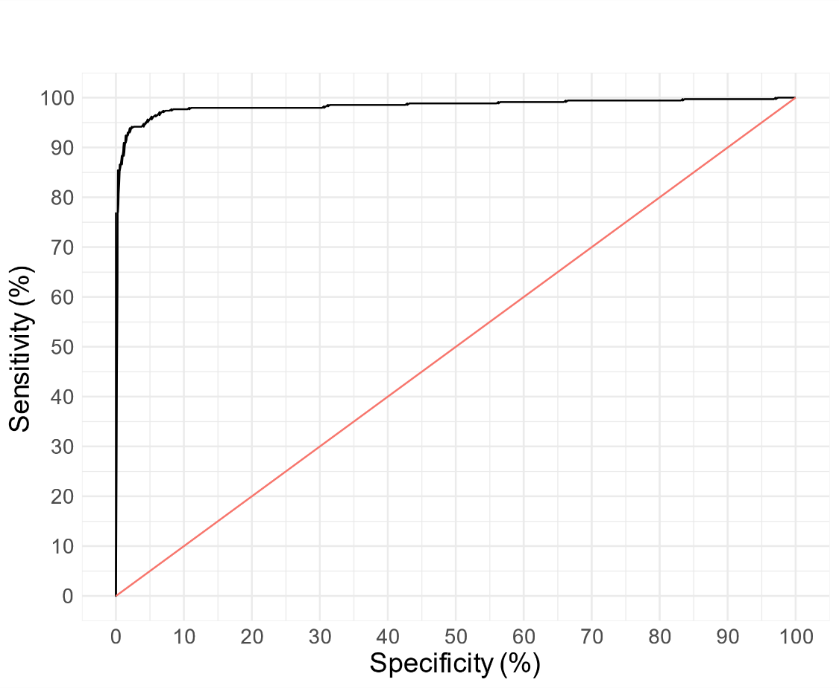


**Supplement Figure 4:** ROC curve analysis of F/F_0_ threshold values

- Area under the curve (AUC) 0.98, p < 0.0001.

Maximal relative threshold value of 1.8 was chosen as this is sensitive and gives a low “false positive” rate (6.7%) and high sensitivity (97.1%) **(Supplement Table 3)**.

|  |  | **Compound** | |  |
| --- | --- | --- | --- | --- |
|  |  | **Buffer** | **NOFQ** | **Total** |
| **Max F/F_0_** | **<1.8** | 320 (TN) | 10 (FN) | **340** |
|  | **>1.8** | 23 (FP) | 333 (TP) | **343** |
|  | **Total** | **343** | **343** |  |

**Supplement Table 3:** Contingency table with threshold for responder cells set at 1.8.

Where maximal F/F_0_ meets or exceeds a threshold of 1.8, individual cells were classified “responders”, the remainder classified “non-responders”. On this basis, using a threshold relative fluorescence of 1.8, the paired responses of cells exposed to 100 μl boluses of N/OFQ (final concentration 10^-6^ M) and Krebs-HEPES Buffer from 5 experiments were classified as responders or non-responders. There was a significantly greater proportion of responsive cells to N/OFQ compared to buffer (χ2, p < 0.05). **(Supplement Figure 5 and Table 4)**.


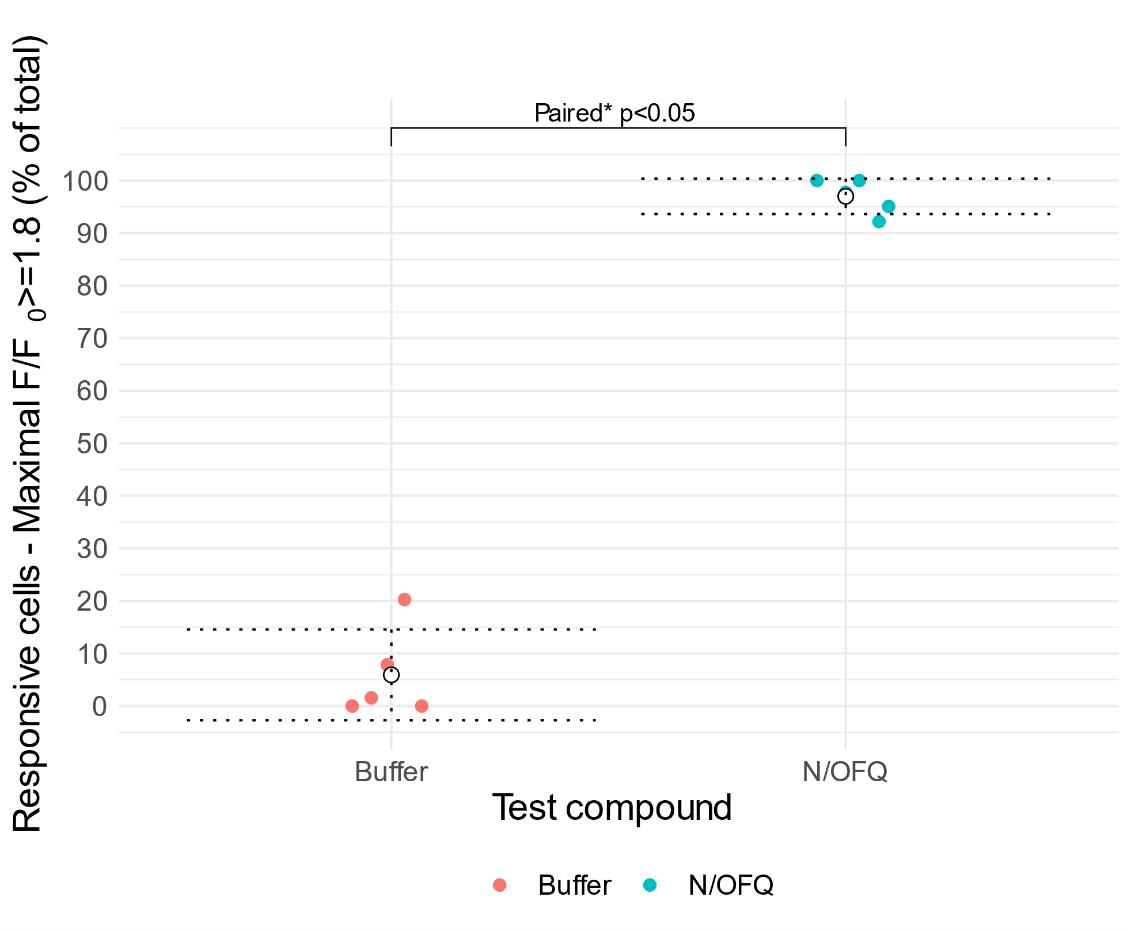


**Supplement Figure 5:** Proportion of cells classified as responders by maximal relative fluorescence following exposure to a bolus addition of 100 μL N/OFQ (final concentration 10^-6^M) and Buffer. - Error bars represent mean ± SD.

|  | **Responders**  **Maximal F/F_0_≥1.8** | **Non-responders**  **Maximal F/F_0_<1.8** |
| --- | --- | --- |
| **N/OFQ**^†^ | 333 | 10 |
| **Buffer** | 23 | 320 |

**Supplement Table 4:** 2 x 2 contingency table showing the significant difference in pooled, paired responses to N/OFQ and buffer - ^†^Significantly greater proportion of responsive cells compared to buffer (χ2, p < 0.05).

**4. CHOwt cells did not respond to N/OFQ and CHO_hNOPGαqi5_ biosensor cells did not respond to fMLP**

In order to confirm transfection of the CHO_hNOPGαqi5_ cells, and to demonstrate that any effect was not due to fMLP, CHO_hNOPGαqi5_ and CHO_WT_ cells were tested in confocal and cuvette based assays. The response from exposure to fMLP 10^-6^ M and N/OFQ 10^-6^ M was ealuated in the confocal and cuvette based assay using the protocol detailed in the main manuscript. Neither cell type showed any significant response to fMLP 10^-6^ M alone, and only the CHO_hNOPGαqi5_ cells showed a response to N/OFQ 10^-6^M. **(Supplement Figure 6)**


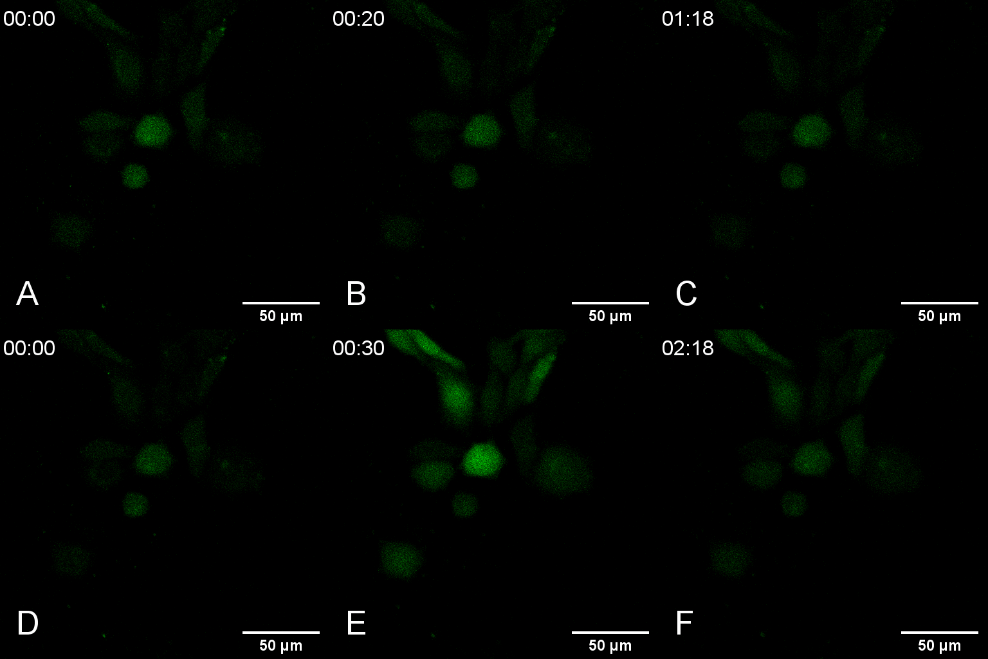


A – Montage – CHO_hNOPGαqi5_ responses to (A-C fMLP, D-F N/OFQ)


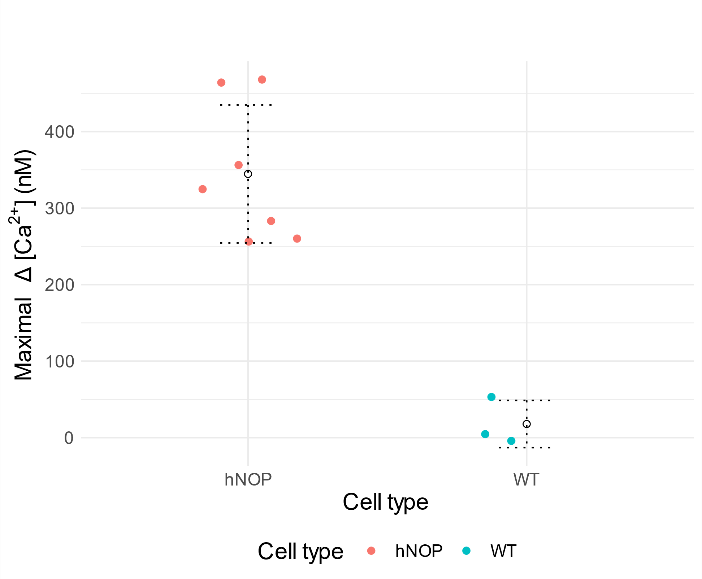


C – Cuvette based fluorimetry responses to N/OFQ


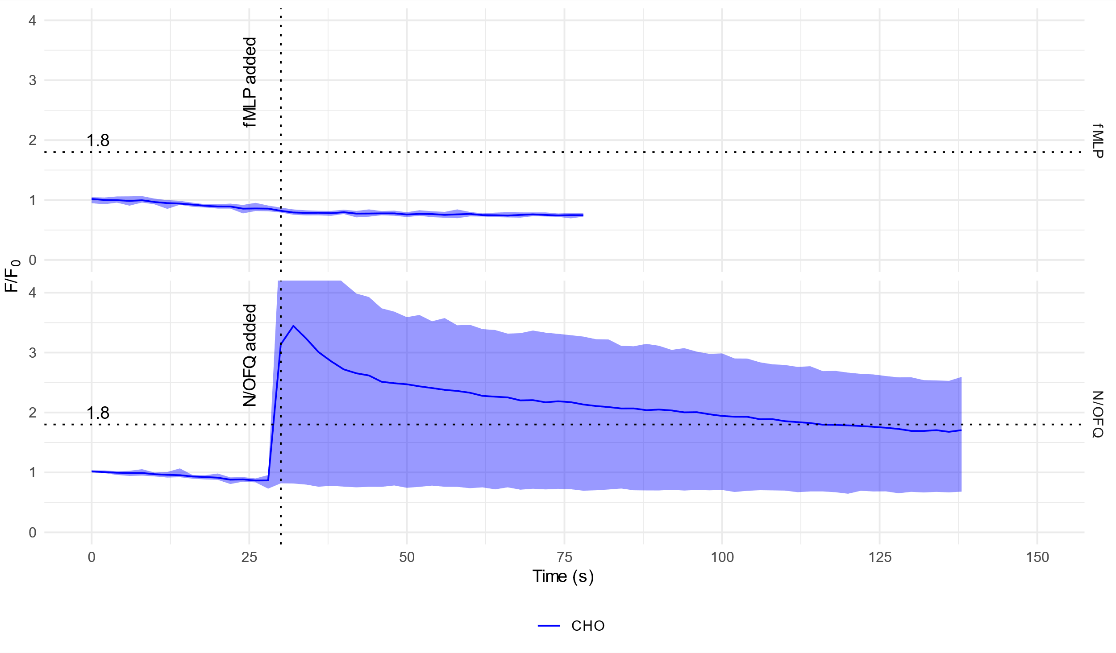
B – Time series CHO_hNOPGαqi5_ response to N/OFQ and fMLP


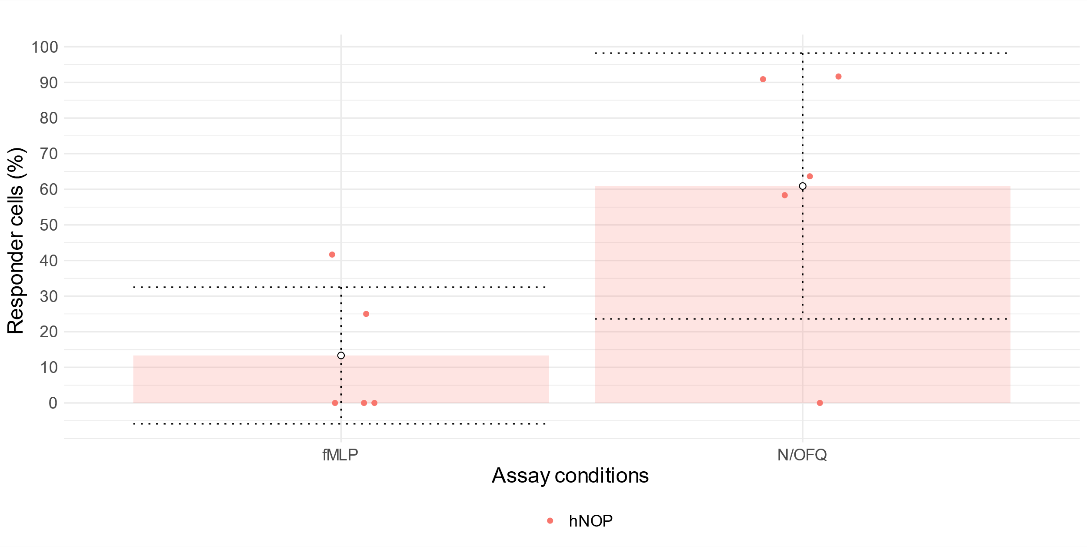


D – Proportion of CHO_hNOPGαqi5_ cells responsive to stated ligands

**Supplement Figure 6:** A- montage of CHO_hNOPGαqi5_ response to fMLP 10^-6^M (A-C), and N/OFQ 10^-6^ M (D-F). A/D Basal, B/E Max, C/F return to basal. B – Time series of A. C – Cuvette based responses of CHO_hNOPGαqi5_ and CHO_WT_ responses to N/OFQ 10^-6^M. D - CHO_hNOPGαqi5_ 10^-6^M pooled confocal responses to fMLP 10^-6^ and N/OFQ 10^-6^ M.

**5. Purinergic antagonist experiment to determine optimal concentration**

The synthetic compound PPADS is known to have antagonist activity at P2X1, P2X2, P2X3, P2X5, P2Y2 and P2Y4 receptors (although not at P2X7). There is a lack of available ligands to block the P2X7 receptor, although oxidised ATP (oATP, Sigma-Aldrich, Dorset, UK) is an irreversible antagonist (with low potency) at this receptor[24]

Therefore, in order to prevent purinergic stimulation upon addition of immune cells to the biosensor, the combination of PPADS and oATP was tested to block both P2X7 and P2Y2 purinergic receptors in the biosensor CHO_hNOPGαqi5_ cells. In order to conserve immune cells, for repetition and to avoid damaging CHO cells by prolonged exposure, the antagonist response was evaluated in the presence of exogenous ATP, and not from immunocyte addition. Antagonist concentrations were determined empirically.

The effect of incubating CHO_hNOPGαqi5_ cells with the nonselective P2 receptor antagonists PPADS (Tocris, 0625) and oATP (Sigma 71997-40-5) in the final concentrations specified (PPADS 5x10^-3^M and oATP 8x10^-4^M) was evaluated against a saturating concentration of 10^-6^ M ATP in the confocal assay, using the protocol described in the main manuscript **(Supplement Figure 7**).


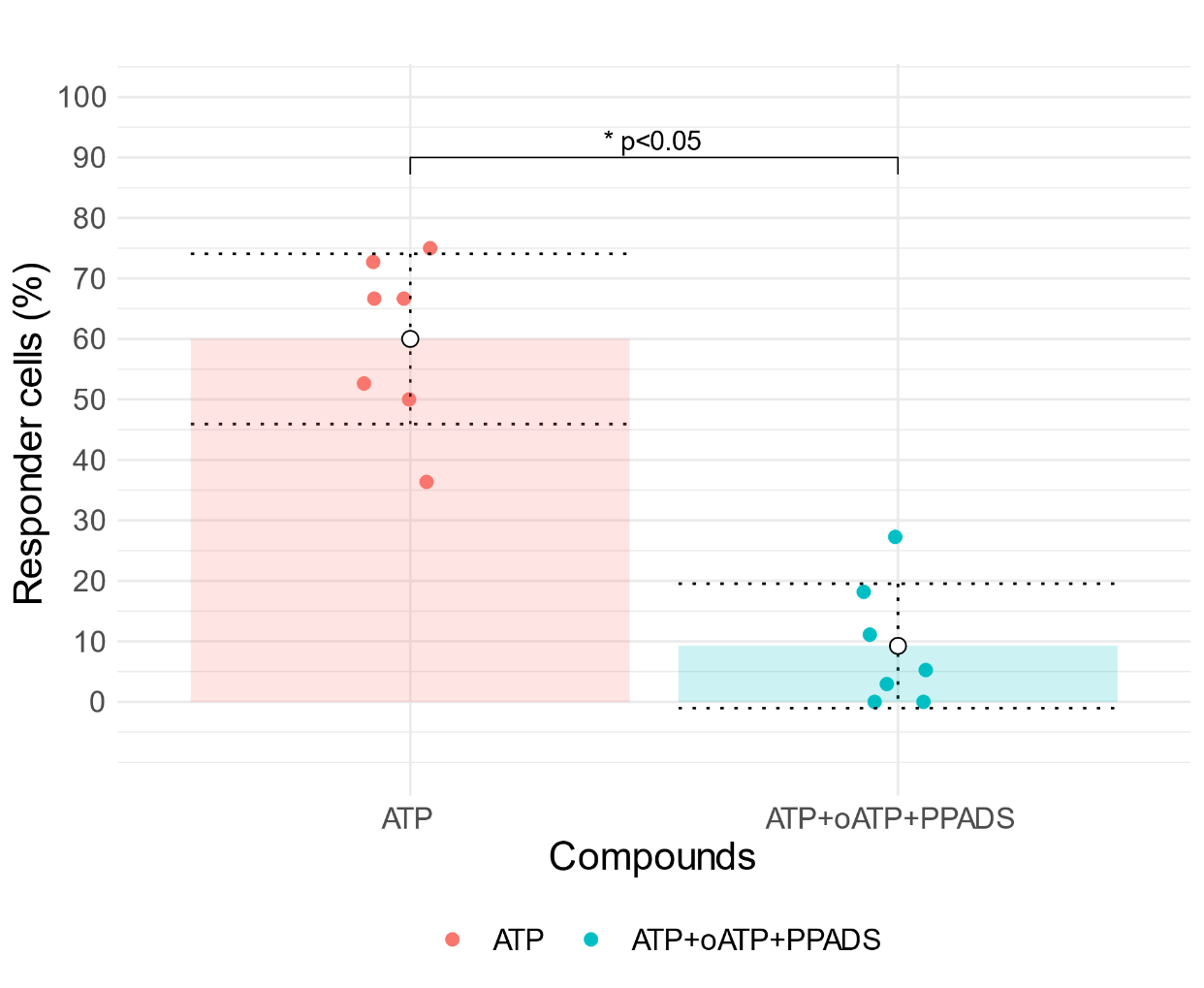


**Supplement Figure 7:** Response of CHO_hNOPGαqi5_ cells to 10^-6^ M ATP in the presence and absence of PPADS and oATP.

**6. Determination of ATP concentrations**

ATP determinations were carried out using the Abcam Luminescent ATP detection kit (Abcam, ab113849) according to the manufacturer’s instructions.

PMNs obtained by Polymorphprep™ based density gradient separation were diluted to 50000, 2500 and 5000 cells per 100 μL of PBS to which 50 μL of the supplied detergent was added for cell lysis. The preparation was agitated at 500 RPM on an orbital shaker for 10 minutes.

Sterile 96 well plates containing luciferin were exposed to cell lysates from 50000, 25000 and 5000 PMNs, and to a serial dilution of ATP (between 10^-8^ M and 10^-4^ M). EOL-1 (Eosinophil Like Cells) cells were used as an example of positive control cells containing ATP.

Luminescence was read following dark adaption for 10 minutes at 37°C on a NOVOstar (BMG Labtech, Aylesbury, UK) microplate fluorimeter, sequentially, for 1 second per well. Duplicate readings were averaged.

The ATP concentration of the cellular samples was determined with reference to the standard curve (**Supplement Figure 8-B**) fitted using GraphPad Prism.

As shown in **Supplement Figure 8**, both PMNs and EOL-1 cells contain ATP, within the 10^-6^ M to 10^-5^ M range; in the absence of purinergic antagonist, endogenous purinergic receptors on the biosensor cells respond to this concentration, indicating the requirement for purinergic antagonism.


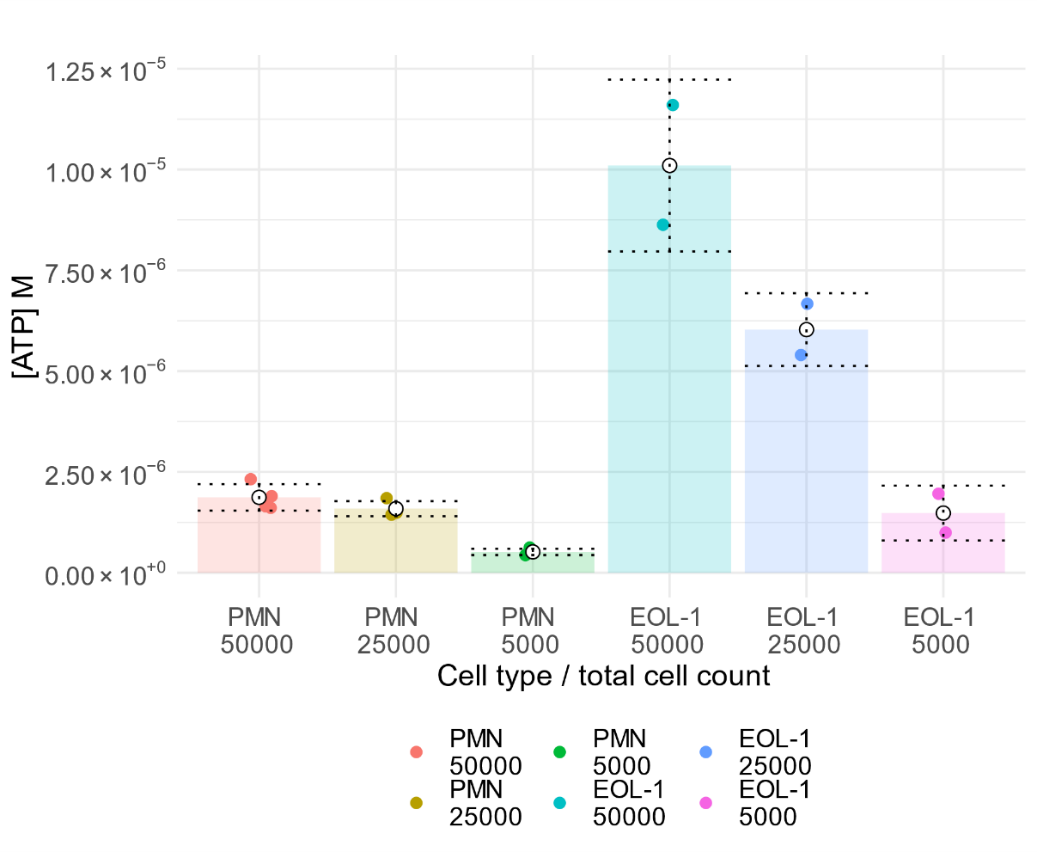


A – ATP concentration released by lysis of cells - Dotted error bars are mean ± SD. PMN: n=4, EOL-1 n=2. Bars represent means


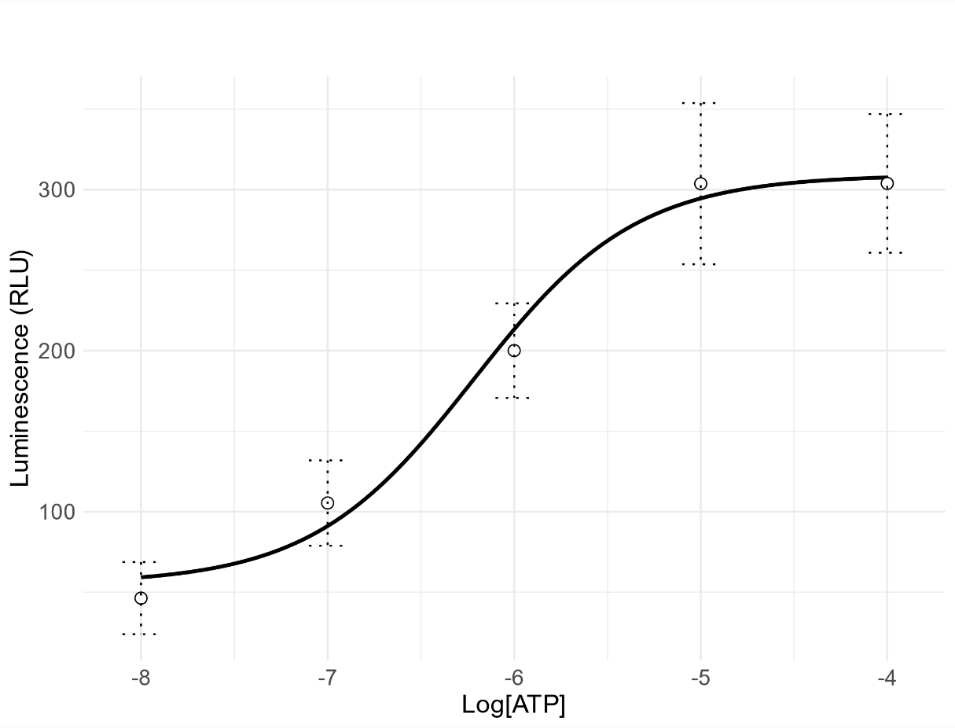


B – Standard curve relating ATP concentration to the luminescence of luciferase (3 point logistical fit)

**Supplement Figure 8:** Total cellular ATP concentration of PMNs and EOL-1 cells as measured by luciferase assay.
